# Secretory expression of cyclohexanone monooxygenase by methylotrophic yeast for efficient omeprazole sulfide bio-oxidation

**DOI:** 10.1186/s40643-021-00430-1

**Published:** 2021-08-27

**Authors:** Ya-Jing Li, Yu-Cong Zheng, Qiang Geng, Feng Liu, Zhi-Jun Zhang, Jian-He Xu, Hui-Lei Yu

**Affiliations:** 1 State Key Laboratory of Bioreactor Engineering, East China University of Science and Technology, Shanghai, 200237 People’s Republic of China; 2 Shanghai Collaborative Innovation Center for Biomanufacturing Technology, East China University of Science and Technology, Shanghai, 200237 People’s Republic of China

**Keywords:** Cyclohexanone monooxygenase, Omeprazole sulfoxide, *Pichia pastoris*, Secretory expression, Asymmetric oxidation

## Abstract

Prochiral pyrmetazole can be asymmetrically oxidized into (*S*)-omeprazole, a proton pump inhibitor that is used to treat gastroesophageal reflux, by an engineered cyclohexanone monooxygenase (CHMO_*Acineto*_*-*Mut) that has high stereoselectivity. CHMO_*Acineto*_*-*Mut is produced by heterologous expression in *Escherichia coli*, where it is expressed intracellularly. Thus, isolating this useful biocatalyst requires tedious cell disruption and subsequent purification, which hinders its use for industrial purposes. Here, we report the extracellular production of CHMO_*Acineto*_*-*Mut by a methylotrophic yeast, *Pichia pastoris,* for the first time. The recombinant CHMO_*Acineto*_*-*Mut expressed by *P. pastoris* showed a higher flavin occupation rate than that produced by *E. coli*, and this was accompanied by a 3.2-fold increase in catalytic efficiency. At a cell density of 150 g/L cell dry weight, we achieved a recombinant CHMO_*Acineto*_*-*Mut production rate of 1,700 U/L, representing approximately 85% of the total protein secreted into the fermentation broth. By directly employing the pH adjusted supernatant as a biocatalyst, we were able to almost completely transform 10 g/L of pyrmetazole into the corresponding (*S*)-sulfoxide, with  >  99% enantiomeric excess.

**Supplementary Information:**

The online version contains supplementary material available at 10.1186/s40643-021-00430-1.

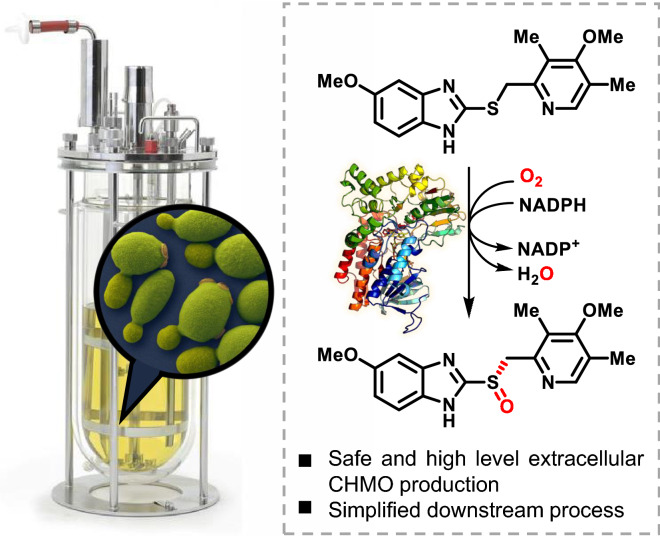

## Introduction

Baeyer–Villiger monooxygenase (BVMO) is a flavin-dependent enzyme that catalyzes the regioselective Baeyer–Villiger oxidation of ketones to the corresponding esters or lactones (Fürst et al. 2019; Romero et al. 2018; de Gonzalo et al. 2010). BVMOs show broad substrate acceptance of aliphatic ketones (Forney and Markovetz 1969; Yu et al. 2018; Fiorentini et al. 2017) and aromatic extended ketones (van Beek et al. 2012; Fraaije et al. 2005), and also catalyze other oxidation reactions, including hydroxylation (Ferroni et al. 2017; Bisagni et al. 2014), epoxidation (Colonna et al. 2002; Rial et al. 2008), and sulfoxidation (Branchaud and Walsh 1985; de Gonzalo et al. 2017). As BVMO catalysis reactions have high enantio-, regio-, and chemo-selectivity, involve a “green” oxidant (oxygen), and utilize the easily recycled NAD(P)H (Mordhorst and Andexer 2020) as the electronic donor, BVMOs are attracting increasing attention for their potential utility in environmentally benign bio-oxidation processes.

Cyclohexanone monooxygenase (CHMO), one of the most well-characterized type I BVMOs (de Gonzalo et al. 2010), was first identified in *Acinetobacter* sp. strain NCIMB 9871 (CHMO_*Acineto*_) (Donoghue et al. 1976). Native CHMO_*Acineto*_ has a strong preference of Baeyer–Villiger oxidation of tetratomic to hexatomic ring ketones (Light et al. 1982). In a recent study, CHMO_*Acineto*_ was subjected to several rounds of directed evolution, resulting in the generation of a mutant (CHMO_*Acineto*_-Mut) with a significant capacity for asymmetric oxidation of pyrmetazole (Bong et al. 2018). This mutant enzyme is used to produce esomeprazole [(*S*)-omeprazole], a popular drug for the clinical treatment of gastroesophageal reflux (Carreno 1995; Matsui et al. 2014). Later studies used genomic mining (Liu et al. 2021; Zhang et al. 2018) or directed evolution to generate several other BVMOs that are capable of transforming sulfides with a “prazole” scaffold (Zhang et al. 2019; Ren et al. 2020; Zhao et al. 2021).

A competitive industrial enzymatic oxidation process always requires a robust biocatalyst, which should be readily available in terms of quantity and have a sufficient activity level. However, both the engineered CHMOs and other native BVMOs are still limited by relatively low activity toward bulky pyrmetazole derivatives. Furthermore, an additional purification step is needed to isolate CHMO_*Acineto*_-Mut, which is produced intracellularly in *Escherichia coli* (Bong et al. 2018). Another issue that should be addressed is the existence of endotoxins in *E. coli* systems, which would bring contamination and affect the quality of the target pharmarco sulfoxides (Hasegawa et al. 1999; Yang and Lee 2008) that it is used to manufacture. Although intracellular expression of CHMOs in generally recognized as safe (GRAS) hosts such as *Saccharomyces cerevisiae* has been shown to be feasible (Chen et al. 1999; Stewart et al. 1996), to date, extracellular production of this enzyme family has not been reported. A safe and efficient heterologous extracellular expression system for CHMO that eliminates the need for cell-breaking and purification steps is therefore needed to achieve large-scale production of high-quality enzymes for industrial applications.

The methylotrophic yeast *Pichia pastoris* (*Komagataella phaffii*) is an established, Food and drug administration (FDA) approved, safe and highly competitive extracellular expression host that secretes large amounts of heterologous proteins and only low amounts of endogenous proteins (Karbalaei et al. 2020; Cereghino and Cregg 2000). Here, we report for the first time extracellular expression of CHMO_*Acineto*_-Mut by *P. pastoris*. The CHMO_*Acineto*_-Mut—containing culture broth was used directly to achieve efficient enzymatic synthesis of (*S*)-omeprazole.

## Experimental

### Chemicals and enzymes

All sulfides, sulfoxides, and sulfones were kindly provided by Aosaikang Pharmaceutical Co., (Nanjing, China). Primer STAR HS and restriction enzymes (*Eco*R I, and *Not* I), and T_4_ DNA ligase were purchased from Takara Bio-technology Co., (Dalian, China). Primers were synthesized by Generay Biotech Co., (Shanghai, China). Unless otherwise stated, all other chemicals and reagents used in this work were obtained commercially and were of reagent grade.

### Strains, plasmids and media

Strains of *E. coli* BL21 (DE3) and *E. coli* DH5α were purchased from TransGen Biotech Co., Ltd. (Beijing, China). *E. coli* DH5α was used for the construction of recombinant plasmids. The *E. coli* strain BL21 (DE3) and the plasmid pET-28a(+) were used for protein expression. *Pichia pastoris* X33 and pPICZαA/pGAPZαA were used for the secretory expression of CHMO_*Acineto*_-Mut.

Luria broth medium (LB, 1% tryptone, 0.5% yeast extract, 1% NaCl), LBK (supplied with kanamycin), Yeast extract—peptone dextrose medium (YPD, 1% yeast extract, 2% peptone and glucose), YPDZ (supplied with zeocin), buffered glycerol—complex medium (BMGY, 1% yeast extract, 2% peptone, 100 mM potassium phosphate (pH 6.0), 0.4 ppm of biotin, 1.34% yeast nitrogen base without amino acids, 1% glycerol), buffered methanol—complex medium (BMMY, 1% yeast extract, 2% peptone, 200 mM potassium phosphate (pH 6.0), 0.4 ppm of biotin, 1.34% yeast nitrogen base, 1% methanol), fermentation basal salts medium (BSM: phosphoric acid (85%, 21 mL/L), CaSO_4_ (0.9 g/L), K_2_SO_4_ (14.3 g/L), MgSO_4_ (6.0 g/L), potassium hydroxide (3.3 g/L) and glycerol (40 g/L)), after autoclaved, 8 mL/L of PTM1 solution was supplied and the pH of the medium was adjusted to 6.0 by ammonia, PTM1 trace salts solution: biotin (0.2 g/L), CuSO_4_·5H_2_O (6.0 g/L), KI (0.09 g/L), MnSO_4_·H_2_O (3 g/L), Na_2_MoO_4_·2H_2_O (0.2 g/L), H_3_BO_3_ (0.02 g/L), CoCl_2_·6H_2_O (0.9 g/L), ZnSO_4_·7H_2_O (42.2 g/L), concentrated H_2_SO_4_ (5 mL/L), FeSO_4_·7H_2_O (65 g/L). Glycerol feeding medium: 50% glycerol supplied with 12 mL/L of PTM1 trace salts solution. Methanol feeding medium: neat methanol supplied with 12 mL/L PTM1 trace salts solution. Bacterial fermentation medium: 0.5% peptone, 0.5 yeast extract, 0.5% glycerol, 0.9% Na_2_HPO_4_·12H_2_O, 0.07% Na_2_SO_4_, 0.34% KH_2_PO_4_, 0.025% MgSO_4_, 0.27% NH_4_Cl. Complex feeding medium: 6% peptone, 6% yeast extract, 25% glycerol.

### Expression and purification of CHMO_*Acineto*_-Mut expressed by *E. coli* BL21 (DE3)

The sequence of the engineered CHMO_*Acineto*_-Mut gene was designed with a histidine tag at the *N*-terminal, synthesized and subsequently cloned into pET-28a(+) by Genscript Biotech (Nanjing) Co., Ltd. (Nanjing, China). Transformants were cultured for 12 h in test tubes containing 4 mL of LB medium with 50 μg/mL kanamycin at 37 °C and 180 rpm, and then 1 mL of the culture was inoculated into 100 mL of fresh LB medium containing 50 μg/mL kanamycin. After cultivation at 37 °C, 180 rpm for 2.5 h, isopropyl-*β*-*D*-thiogalactoside (IPTG) and vitamin B solutions were added to a final concentration of 0.2 mM, and 50 mg /L, respectively. Induction was started when the optical density (OD_600_) of the *E. coli* cells arrived at 0.6–0.8, and further proceeded for 20 h at 16 °C, 180 rpm. The induced cells were harvested by centrifugation and lysed by ultra-sonication, and the cell lysate was centrifuged at 10,000×*g* and 4 °C for 30 min. The supernatant was then loaded onto a HisTrap HP (GE, USA) column which was pre-equilibrated with Ni–NTA buffer A (potassium phosphate, 20 mM, pH 7.4; sodium chloride, 500 mM; 2-mercaptoethanol, 5 mM). Samples were eluted by gradient imidazole by employing Ni–NTA buffer B (Ni–NTA buffer A containing 500 mM of imidazole). Elution fractions which contained highly pure CHMO_*Acineto*_-Mut were pooled, desalted with Ni–NTA buffer C (potassium phosphate, 20 mM, pH 7.4; sodium chloride, 150 mM; dithiothreitol, 1 mM) and flash frozen in liquid nitrogen, stored at − 80 °C for further analysis.

### Cloning and expression of the CHMO_***Acineto***_-Mut gene in ***P. pastoris*** X33

The CHMO_*Acineto*_*-*Mut gene was amplified using pET-28a(+)-CHMO_*Acineto*_*-*Mut as the template by PCR under the following conditions: pre-denaturation at 95 °C for 3 min; 29 cycles at 98 °C for 10 s, 55 °C for 15 s, and 72 °C for 105 s; followed by a final extension at 72 °C for 10 min. The primers used in this study were shown in Additional file 1: Table S1. The PCR product was ligated into pPICZαA/pGAPZαA and then cloned into *E. coli* DH5α. About 5 μg plasmids were linearized by *Sac* I for 6 h and recovered with PCR purification Kit (Aidlab Biotechnologies Co., Ltd., Beijing, China), and then electrotransformed into *P. pastoris* X33 with a Micropulser (BioRad, Hercules, CA, USA). Transformants were selected on YPD (supplemented with zeocin) plates after incubation for 48 h at 30 °C.

Positive yeast transformants were cultured for 24 h in test tubes containing 4 mL of YPD medium with 100 μg/mL zeocin at 28 °C and 200 rpm, and then 1 mL of the culture was inoculated into 100 mL of BMGY medium containing 100 μg/mL ampicillin. After cultivation at 28 °C, 200 rpm until optical density of the yeast cells arrived at 1–2, cells were resuspended in BMMY medium and further induced at 28 °C, 200 rpm for 96 h. Neat methanol (1%, v/v) was added into the medium at further 24, 48, 72 and 96 h, respectively.

### Purification of CHMO_***Acineto***_-Mut expressed in yeast

After cultivation, cells were removed from the induced yeast fermentation broth by centrifugation at 6,000 × *g*, 4 °C for 40 min. About 500 mL fermentation clear broth was subjected to microfiltration (0.45 μm), concentrated by ultrafiltration on a Labscale™ Tangential Flow Filtration System (Merck Millipore, German) equipped with a 30 kDa cut off module (Pellicon® XL, 50 cm^2^, Millipore, Germany). The concentrated broth was diluted and concentrated twice with Ni–NTA buffer A. Samples were centrifugated at 10,000 × *g*, 4 °C for 30 min to remove deactivated proteins, then further purified by Ni^2+^-affinity chromatography (Ren et al. 2020) and flash frozen in liquid nitrogen, stored at ‒80 °C for further analysis.

### High level production of CHMO_***Acineto***_-Mut by high cell density fermentation

#### CHMO_***Acineto***_-Mut-_*P*_

Single recombinant yeast colony was isolated on YPDZ agar plates and pre-cultured (200 rpm, 30 °C) in 200 mL liquid YPD medium which contained ampicillin (100 μg/mL) until the optical density of the cells arrived at 2.0, then inoculated into a 5-L bioreactor (BXBIO, shanghai, China) which contained 1.8 L BSM medium. The fermentation was carried out at 28 °C, DO  >  20%, and pH 6.0. After about 18 h of the fermentation, the DO value was suddenly rebounded to 100%, indicating the consumption of glycerol in the initial medium, and then glycerol feeding was started until an OD_600_ of about 200. A starvation was kept for 1 h to make sure the metabolism intermediates of glycerol were consumed. Then methanol induction was started with an initial rate of 4 mL/h during the first 4 h to make the yeast adapt to methanol before being stepped to a higher rate of about 18 mL/h in 12 h. After the fermentation, cells were removed and the yeast secretion supernatant was kept on ice for further analysis.

#### CHMO_***Acineto***_-Mut-_***E***_

Single recombinant *E. coli* colony was isolated on LBK agar plates and precultured (180 rpm, 37 °C) in 300 mL liquid bacterial fermentation medium supplied with kanamycin (50 μg/mL) in shake flask until the optical density arrived at 1.5, then inoculated into 5-L bioreactor which contained 2.7 L bacterial fermentation medium. The fermentation was carried out at 37 °C, DO > 20%, and pH 7.0. After 4 h, the complexed feeding medium was supplied with a constant rate of 25 mL/h. Cells were induced when the OD_600_ arrived about 8.0 by adding IPTG stock solution to a final concentration of 200 μM at 25 °C.

### Protein assays and determination of the FAD occupation rate

The concentration of CHMO_*Acineto*_-Mut was determined by Bradford method, using bovine serum albumin (BSA) as the standard. UV–Vis scanning was performed on a Molecular Devices SpectraMax M2 Microplate Reader (USA). Determination of FAD occupation was modified from Fraaije’s work (Fraaije et al. 2005), purified CHMO_*Acineto*_-Mut samples were diluted to 5 mg/mL by denature buffer (potassium phosphate, 20 mM, pH 8.0; NaCl, 150 mM; DTT, 1 mM; SDS, 1%, w/v) and incubated at 95 °C for 5 min, then the absorbance was analyzed in the wavelength range of 280–447 nm, respectively.

### Determination of intracellular FAD content of *E. coli* and *P. pastoris*

At the scheduled time, cells (*E. coli* or *P. pastoris*, 132 OD) were resuspended in 100 mM potassium phosphate buffer (pH 9.0, 0.5 mL), the suspension was subjected to agitation at 1500 rpm, 4 °C for 30 min by 200 mg glass beads (Φ 1 mm). A clear cell lysate was obtained by centrifugation of the sample at 10,000×*g*, 5 min. Samples were further incubated at 95 °C for 10 min and centrifugated again at 10,000×*g* for 10 min to remove the protein. The resultant supernatant was subjected to HPLC analysis by a SHIMADAZU LC2010A system equipped with a C18 column (250 mm × 4.6 mm, 10 μm particle size, DEAIC), under 30 °C, 447 nm, using methanol/water (6/4, v/v, 0.6 mL min^−1^) as the mobile phase (*t*_R_ FAD  =  4.531 min).

### Glycosylation characterization of CHMO_***Acineto***_-Mut

Purified CHMO_*Acineto*_-Mut-_*E*_ and CHMO_*Acineto*_-Mut-_*P*_ was diluted with denature buffer to 1 mg/mL and incubated at 95 °C for 10 min, then cooled to room temperature. 10 μg of the sample was mixed with 10 μg of the *Sp*Endo H and incubated at 37 °C for 1 h. Expression, purification of *Sp*Endo H was conducted according to our previous work (Zheng et al. 2020). Samples were further analysed by SDS-PAGE.

### Bio-asymmetric oxidation of pyrmetazole

After cultivation, cells were removed by centrifugation at 6000×*g*, 4 °C for 30 min. The pH value of the resulting fermentation clear broth was adjusted to 8.0 by slowly dropping 1 M K_2_CO_3_ aqueous solution at 0 °C. For 10-mL scale reactions, the reactions were performed in closed 50 mL shake flask which contained 100 mM potassium phosphate buffer (pH 8.0), 0.2 mM NADP^+^, lyophilized *Bst*FDH preparation. The reaction was initiated by adding 1 mL pyrmetazole methanol stock solution and shaken at 25 °C, 180 rpm. For 0.6 L scale bio-oxidation reaction, the reaction was performed in a jacked 1 L fermenter, and the reaction mixture was scaled up. At the schedule time, 100 μL of the reaction mixture was taken, extracted by ethyl acetate (0.5 mL) and analyzed by chiral HPLC (Xu et al. 2020).

### Purification and characterization of esomeprazole sodium salt

Purification of the esomeprazole sodium was conducted according to the modified procedure by Xu et al. (2020). After the reaction was finished, the pH was adjusted to 11 by NaOH and subjected to centrifugation at 6000×*g* for 0.5 h. The supernatant was neutralized by acetic acid to pH 7, then extracted by ethyl acetate. The combined organic layers were dried over anhydrous Na_2_SO_4_, concentrated under reduced pressure. The resultant residual was dissolved in ethanol, added with 1 equiv. of NaOH powers, and the resulting solution was crystalized in cold ethanol/methyl-tert-butyl-ester to afford esomeprazole sodium salt.

## Results and discussion

### Construction of recombinant ***P. pastoris*** for secretory expression of CHMO_***Acineto***_-Mut

**Fig. 1:**
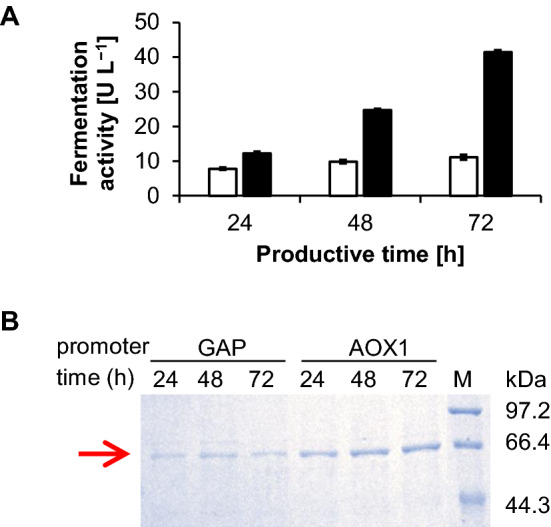
Initial assessment for the best CHMO_*Acineto*_-Mut extracellular production in shake flask fermentations. **A** Fermentation activity of CHMO_*Acineto*_-Mut under *AOX*1 promoter (■) and *GAP* promoter (□) with different fermentation time. **B** SDS-PAGE analysis of the extracellular expression of CHMO_*Acineto*_-Mut by *P. pastoris* X33 under the control of *GAP* and *AOX*1 promoters, respectively. The red arrow indicated CHMO_*Acineto*_-Mut

First, expression of CHMO_*Acineto*_-Mut under the control of the strong, tightly regulated *AOX*1 promoter and the constitutive *GAP* promoter was compared. To do this, the gene encoding CHMO_*Acineto*_-Mut (Bong et al. 2014) was ligated into pPICZαA and pGAPZαA, respectively. Next, the expression cassettes were integrated into the *P. pastoris* X33 (His^+^, Mut^+^) genome via transformation of the linearized plasmids and subsequent homologous recombination. Zeocin-resistant transformants were selected and rescreened by incubating cultures in shake flasks and testing the culture supernatants for pyrmetazole oxidation activity. The fermentation activity of the X33-pGAPZαA-CHMO_*Acineto*_-Mut strains peaked at 10 U/L, even when glucose was supplied (Fig. 1A). In contrast, an approximately 65-*k*Da protein band increased in intensity in parallel with increasing pyrmetazole oxidation activity (from 12 to 41 U/L) when CHMO_*Acineto*_-Mut was expressed under the control of the *AOX*1 promoter (Fig. 1B). Therefore, we selected this strain for further investigation. To identify strains with multiple integration events, X33-pPICZαA-CHMO_*Acineto*_-Mut transformants were grown on YPDZ plates with different zeocin concentrations. The strain that exhibited the greatest zeocin resistance (Additional file 1: Figure S1) and the highest CHMO production was selected for use in all subsequent experiments.

### Characterization of the activity of CHMO_***Acineto***_-Mut expressed by different microbial hosts

To characterize the activity of recombinant CHMO_*Acineto*_**-**Mut secreted by *P. pastoris*, we constructed a *P. pastoris* strain expressing CHMO_*Acineto*_-Mut with a *C*-terminal histidine tag. The tagged CHMO_*Acineto*_-Mut was then purified from the yeast supernatant by ultrafiltration followed by Ni–NTA chromatography. Surprisingly, CHMO_*Acineto*_-Mut expressed by yeast (CHMO_*Acineto*_-Mut-_*P*_) exhibited approximately 4 times greater specific activity than CHMO_*Acineto*_-Mut expressed by *E. coli* (CHMO_*Acineto*_-Mut-_*E*_). Next, we determined the Michaelis–Menten kinetic constants of pyrmetazole oxidation by CHMO_*Acineto*_-Mut-_*P*_ and CHMO_*Acineto*_-Mut-_*E*_ (Table 1) to investigate biochemical differences in CHMO_*Acineto*_-Mut expressed by different hosts. There was no difference between CHMO_*Acineto*_-Mut-_*P*_ and CHMO_*Acineto*_-Mut-_*E*_ in terms of affinity for pyrmetazole; however, CHMO_*Acineto*_-Mut-_*P*_ exhibited a 3.2-fold higher turnover frequency (*k*_cat_) than CHMO_*Acineto*_-Mut-_*E*_. Similar phenomenon was observed in the kinetic profiles of NADPH, indicating that CHMO_*Acineto*_-Mut-_*P*_ has greater catalytic efficiency than CHMO_*Acineto*_-Mut-_*E*_.

**Table 1:** Kinetic profiles of recombinant CHMO_*Acineto*_-Mut expressed in *E. coli* and *P. pastoris* in pyrmetazole oxidation reactions

Enzyme	Pyrmetazole^a^			NADPH^b^		
	*K*_M_ (mM)	*k*_cat_ (s^−1^)	*k*_cat_/*K*_M_ (s^−1^ mM^−1^)	*K*_M_ (mM)	*k*_cat_ (s^−1^)	*k*_cat_/*K*_M_ (s^−1^ mM^−1^)
CHMO_*Acineto*_-Mut-_*E*_	0.028 ± 0.004	0.35 ± 0.02	12.5	(11.2 ± 2.5) × 10^–4^	0.34 ± 0.02	304
CHMO_*Acineto*_-Mut-_*P*_	0.026 ± 0.001	1.12 ± 0.04	43.4	(7.1 ± 1.1) × 10^–4^	0.82 ± 0.02	1154

^a^Kinetic parameters were determined in 0.5 mL reaction scale which contained potassium phosphate (100 mM, pH 9.0), methanol (2%, v/v), various concentrations of pyrmetazole (2–400 μM), NADPH (200 μM) and appropriate amount of CHMO_*Acineto*_-Mut

^b^Reactions were determined by employing pyrmetazole (200 μM) and various concentrations of NADPH (5–200 μM). For detailed analytical conditions, see experimental sections

**Fig. 2:**
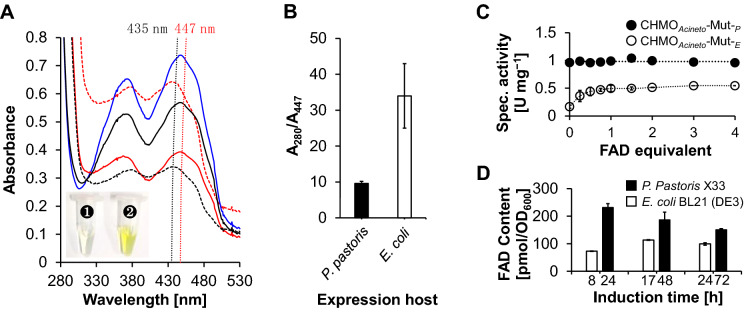
**A** Representative UV-visual spectrum of CHMO_*Acineto*_-Mut-_*P*_ (red dash line), CHMO_*Acineto*_-Mut-_*E*_ (black dash line), free FAD (blue solid line), boiled CHMO_*Acineto*_-Mut-_*P*_ (black solid line) and boiled CHMO_*Acineto*_-Mut-_*E*_ (red solid line); and photograph showing the color of CHMO_*Acineto*_-Mut-_*E*_ , CHMO_*Acineto*_-Mut-_*P*_  with the same concentration (5 mg/mL). **B** Calculated A_280_/A_447_ value of CHMO_*Acineto*_-Mut expressed by different hosts according to the UV–Vis spectrum analysis. **C** Specific activity of CHMO_*Acineto*_-Mut-_*P*_ (□) and CHMO_*Acineto*_-Mut-_*E*_ (○) supplied with different amounts of free FAD. **D** Intracellular FAD constitution of *P. pastoris* X33 and *E. coli* BL21 (DE3) during the expression of CHMO_*Acineto*_-Mut

In parallel, we noted that CHMO_*Acineto*_-Mut-_*P*_ showed more intense yellow color during the purification process, albeit when it was diluted to the same protein concentration of CHMO_*Acineto*_-Mut-_*E*_. Because this enzyme belongs to the type I BVMO superfamily, it is functionally dependent on flavin adenine dinucleotide (FAD), which is the source of the yellow color of the enzyme. The UV-visual spectrum profile of CHMO_*Acineto*_-Mut-_*P*_ and CHMO_*Acineto*_-Mut-_*E*_ at a concentration of 5 mg/mL was therefore analyzed. As expected, the characteristic twin peaks of isoalloxazine (FAD) in CHMO_*Acineto*_-Mut-_*P*_ were significantly higher than those of CHMO_*Acineto*_-Mut-_*E*_, indicating higher FAD occupation (Fig. 2A). Given that FAD absorbs light at a wavelength of 447 nm (Fraaije et al. 2005), we determined the A_280_/A_447_ of the two denatured CHMOs and found that CHMO_*Acineto*_-Mut-_*P*_ had about 3.5-fold higher FAD occupation than CHMO_*Acineto*_-Mut-_*E*_ (Fig. 2B), which is in consist with the higher *k*_cat_ value of CHMO_*Acineto*_-Mut-_*P*_ than CHMO_*Acineto*_-Mut-_*E*_.

Next, we added different amounts of free FAD to the activity determination system to determine whether it affected the activity of the heterologously expressed proteins. As expected, a stepwise increase in enzyme activity (up to about 1.5-fold, Fig. 2C) that correlated with increasing FAD concentrations was observed for CHMO_*Acineto*_-Mut-_*E*_ but not CHMO_*Acineto*_-Mut-_*P*_. Interestingly, during the induction phase of fermentation, yeast contained more intracellular FAD than *E. coli* (Fig. 2D), which indicates that using yeast as the expression host results in a greater FAD supply, as well as greater incorporation of FAD into the recombinant CHMO_*Acineto*_-Mut. These results demonstrated that the eukaryotic *P. pastoris* is a better system for expressing FAD-dependent enzymes than the prokaryotic *E. coli*.

Next, we assessed *N*-glycosylation of CHMO_*Acineto*_-Mut-_*P*_ by digesting with *Streptomyces plicatus* endoglycosidase H (Additional file 1: Figure S3). The results confirmed that *N*-glycosylation of CHMO_*Acineto*_-Mut-_*P*_ occurred only at the *N*-glycosylation motif (N-X-T/S) at Asn249. However, glycosylation did not change the thermostability of CHMO_*Acineto*_-Mut. Furthermore, the *T*_m_ values for CHMO_*Acineto*_-Mut-_*P*_ and CHMO_*Acineto*_-Mut-_*E*_ were both 60 °C, as determined by the *Thermo*FAD method (Additional file 1: Figure S4; Forneris et al. 2009).

### Production of CHMO_***Acineto***_-Mut via high-density fermentation on a 5-L scale

**Fig. 3:**
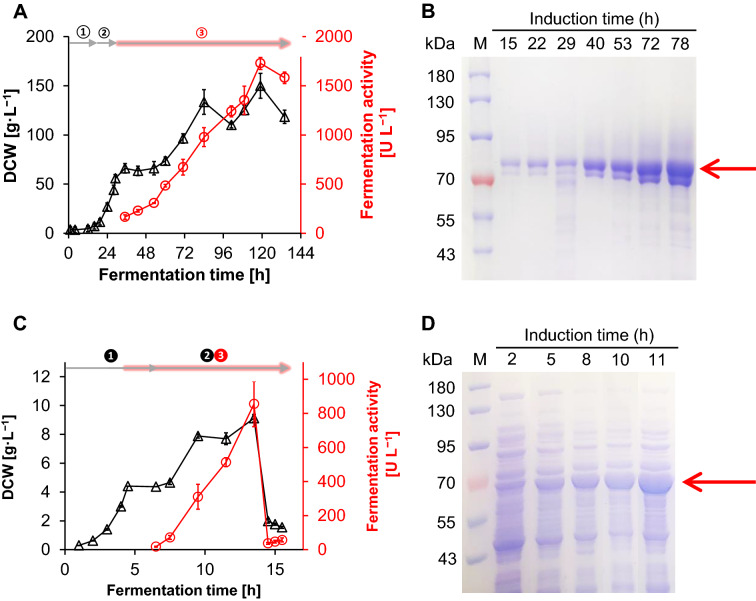
Time course of high-cell-density fermentation of strain X33-pPICZαA-CHMO_*Acineto*_-Mut (**A**) or BL21 (DE3)-pET-28(a) + -CHMO_*Acineto*_-Mut (**C**) in 5 L fermentator and the SDS-PAGE analysis of the yeast fermentation clear broth (**B**) or the cell-free extract of the *E. coli* (**D**) during the induction processes. For the *P. pastoris* fermentation, the time course was divided into the glycerol consumption , glycerol feeding  and methanol induction ; and corresponding to the glycerol consumption , glycerol feeding  and IPTG induction  for *E. coli*. lane M, protein marker; the red arrow highlighted the band of CHMO_*Acineto*_-Mut

Production of CHMO_***Acinet****o*_-Mut by either recombinant *P. pastoris* or *E. coli* in a 5-L fermenter was subsequently compared. Pyrmetazole oxidation activity was observed immediately after induction with methanol in the yeast fermentation process (Fig. 3A). Enzyme production by recombinant *P. pastoris* peaked at 1728  ±  63 U/L, which is almost 2.8-fold that seen with recombinant *E. coli,* after 103 h of methanol feeding (Fig. 3C). The specific activity of purified CHMO_*Acineto*_*-*Mut-_*P*_ was 0.95  ±  0.01 U/mg, which suggests that the concentration of CHMO_*Acineto*_*-*Mut-_*P*_ in the yeast fermentation broth was greater than 1.6 g/L. The high extracellular expression level of CHMO_*Acineto*_*-*Mut-_*P*_ was also confirmed by 10% SDS-PAGE analysis (Fig. 3B). The most intense protein band at about 75 kDa represented CHMO_*Acineto*_-Mut-_*P*_, whereas the content of other proteins in the supernatant was negligible. SDS-PAGE analysis of cell-free *E. coli* extracts indicated that CHMO_*Acineto*_-Mut-_*E*_ accounted for only approximately 50% of the total protein content (Fig. 3D). Besides, *E. coli* BL21(DE3) strain usually suffered from cell autolysis (Wagner et al. 2008; Narayanan et al. 2008), which is obscure to control the right chance for cell harvest at the fermentation anaphase. Recombinant *P. pastoris* therefore exhibited great operation stability for fermentation process. More importantly, the cost of the raw materials needed for CHMO_*Acineto*_-Mut-_*P*_ production was only 28% of that need to produce CHMO_*Acineto*_-Mut-_*E*_ (Table 2). This was primarily due to the markedly simpler growth medium composition, and further emphasizes the technical and economic advantages of employing *P. pastoris* in the production of CHMO_*Acineto*_-Mut.

**Table 2:** Comparison of CHMO_*Acineto*_-Mut production in *E. coli* and *P. pastoris*

Strain	Fermentation time (h)	Peak activity (U/L)	Productivity (U L^−1^ h^−1^)	CHMO constitution (%)	Cell disruption	Cost^a^ (CNY/kU)
*P. pastoris* X33	119 (103)^b^	1728 ± 63	14.5	85	N	0.66
*E. coli* BL21 (DE3)	15.5 (10.5)^b^	855 ± 133	55.1	50	Y	2.32

^a^For calculation details of the cost for fermentation and preparation of CHMO_*Acineto*_-Mut from different host, see Additional file 1: Table S2

^b^Induction time

### Asymmetric bio-oxidation of pyrmetazole by employing yeast secretion

One of the most important advantages of using methylotrophic yeast as an expression system for biotransformation is the direct use of yeast secretion as the catalyst (Zheng et al. 2020; Qian et al. 2014). Bio-asymmetric synthesis of (*S*)-omeprazole by utilizing CHMO_*Acineto*_-Mut-_*P*_ in the yeast secretion was then conducted. The *Burkholderia stabilis* 15516 formate dehydrogenase (*Bst*FDH) (Xu et al. 2020; Hatrongjit and Packdibamrung 2010) was coupled for the regeneration of the cofactor NADPH. In the initial set of experiments, moderate rates (65–86%) of pyrmetazole conversion were observed within 17 h (Table 3, entries 1, 2). Considering the low level of soluble oxygen in aqueous solution is usually the limitation especially for the enzymatic oxidation reactions. The reaction was then performed under an oxygen atmosphere with an oxygen balloon to supply oxygen. As expected, when additional oxygen was provided, 10 g/L of substrate was completely transformed into the target product sulfoxide (Table 3, entry 3). However, further increase the substrate loading to 20 g/L resulted to only 82% conversion after 17 h (Table 3, entry 4), which might be attributed to the mass transfer problem raised by the high hydrophobicity of both pyrmetazole and the corresponding sulfoxide. Based on the optimal conditions determined using a 10-mL shaker flask, the bio-asymmetric sulfoxidation reaction was scaled up to 0.6 L (Table 3, entry 4, also see Additional file 1: Figure S7). After 26 h, near-complete oxidation (97% conv.) was achieved, with excellent optical purity (> 99% *ee*). During extraction of the sulfoxide product from the aqueous phase, we noted that there was a remarkable decrease in CHMO_*Acineto*_-Mut-_*P*_ emulsion compared with the CHMO_*Acineto*_-Mut-_*E*_ crude extract (Additional file 1: Figure S6), which we attributed to the lower concentrations of endogenous protein and nucleic acids in the yeast supernatants. Ultimately, a 73% isolation yield for the crude product was achieved, which can be refined further for pure esomeprazole sodium salt.

**Table 3:** Optimization of bio-oxidation of pyrmetazole by employing recombinant yeast secretion^a^
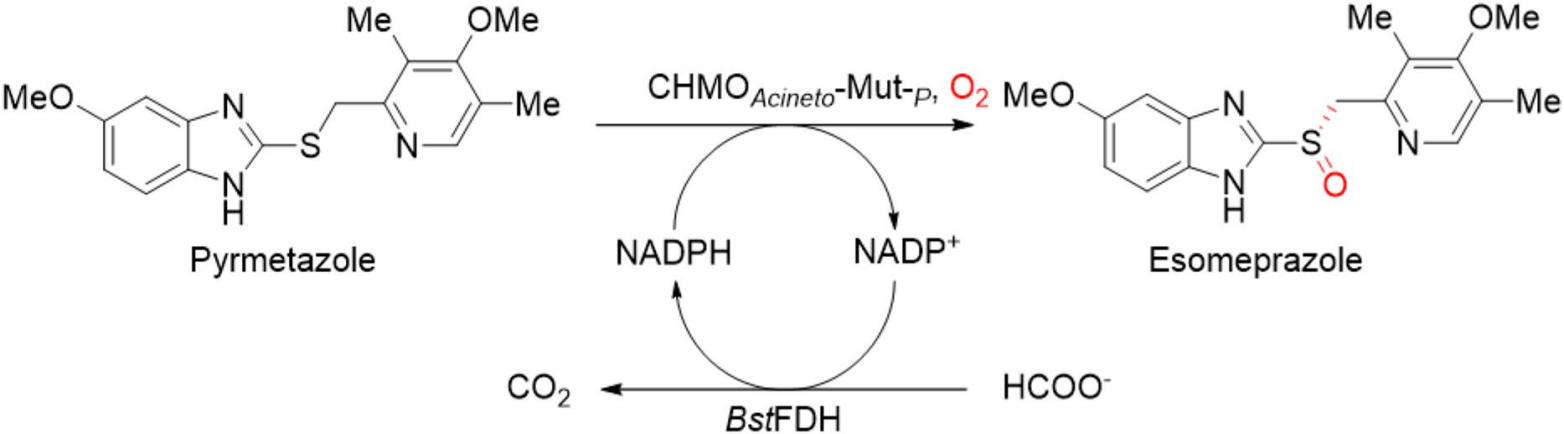

Entry	CHMO_*Acineto*-_Mut-_*P*_/*Bst*FDH loading (U/U)	Reaction scale (mL)	Conversion (%)^c^				*ee* (%)^c,g^
			3 h	9 h	17 h	26 h	
1	1.5/3.7	10	15	41	65	nd^d^	> 99
2^b^	1.5/3.7	10	24	59	86	nd^d^	> 99
3	4.5/11	10	32	78	> 95	nd^d^	> 99
4^b^	4.5/11	10	49	98	> 99	nd^d^	> 99
5^b^^,e^	4.5/11	10	36	67	82	nd^d^	> 99
6^e,f^	450/450	600	10	30	72	97	> 99

^a^Reactions were performed in 50-mL shake flask, the reaction mixture contained potassium phosphate buffer (100 mM, pH 8.0), 100 mM sodium formate, lyophilized FDH preparation, 0.2 mM NADP^+^, and neutralized yeast fermentation clear broth (adjust pH to 8.0 by using 1 M K_2_CO_3_ aqueous solution), methanol (10%, v/v) and pyrmetazole (10 g L^−1^). The reaction mixture was incubated at 180 rpm, 25 °C

^b^Reactions were performed under oxygen atmosphere (O_2_-balloon)

^c^Determined by chiral HPLC

^d^Not determined

^e^The pyrmetazole loading was increased to 20 g L^−1^

^f^Reaction was performed in a 1-L jacked fermenter and stirred at 150 rpm and bubbled with air (0.5 vvm)

^g^The *ee* value was determined at 9 h

## Conclusions

In summary, we demonstrated for the first time extracellular production of CHMO_*Acineto*_-Mut (a type I BVMO), with excellent yield and good purity, using a *P. pastoris* X33 Mut^+^ expression system. When the neutralized yeast supernatants were used directly to catalyze asymmetric Kagan − Sharpless − Pitchen sulfoxidation of 10 g/L pyrmetazole, a satisfactory product *ee* and conversion rate were achieved. Moreover, using this *P. pastoris* expression system obviated two major hurdles associated with CHMO in *E. coli,* namely the insufficient FAD supply and the need for a tedious cell-disruption step. As flavin-dependent enzymes are widely used in the pharmaceutical, agricultural, food production, and synthetic chemistry industries (Baker Dockrey and Narayan 2019), *P. pastoris* may be a suitable host for the production of flavin-dependent enzymes for a wide range of industrial purposes. Thus, this work provides a valuable example of how efficient extracellular production of flavin-dependent biocatalysts can be achieved.

### Supplementary Information

**Additional file 1: Table S1. **Primers used in this study. **Table S2.** The cost of raw materials for CHMO_*Acineto*_-Mut preparation using different expression host. **Figure S1.** Shake flask fermentation activity of high-copy strain screening. **Figure S2.** Kinetic curves of CHMO_*Acineto*_*-*Mut*-*_*P*_ and CHMO_*Acineto*_*-*Mut*-*_*E*_ toward pyrmetazole (A) and NADPH (B). **Figure S3.** SDS-PAGE of deglycosylated CHMO_*Acineto*_-Mut by *Sp*Endo H. **Figure S4.** Melting curves of CHMO_*Acineto*_*-*Mut*-*_*E*_ and CHMO_*Acineto*_*-*Mut*-*_*P*_ determined by *Thermo*FAD analysis. **Figure S5.** Photograph show of the ethyl acetate extraction of the esomeprazole in the aqueous phase of the reaction mixture (A) CHMO_*Acineto*_*-*Mut*-*_*E*_. (B) CHMO_*Acineto*_*-*Mut*-*_*P*_. **Figure S6.** Enzymatic oxidation reaction of pyrmetazole performed in 0.6 L scale and the isolation of esomeprazole sodium. **Figure S7.** Representative HPLC spectrum of FAD analysis of intracellular constituent. **Figure S8.** Representative HPLC spectrum of enzymatic esomeprazole synthesis. **Figure S9.** NMR spectrums of esomeprazole sodium salt. **Figure S10.** LR-MS spectrums of esomeprazole sodium salt.

## Data Availability

All data generated or analyzed during this study are included in this article and the supplementary information file.

## Abbreviations

*AOX*1: Alcohol oxidase 1 promoter
*Bst*FDH: Formate dehydrogenase from *Burkholderia stabilis* 15516
BVMO: Baeyer–Villiger monooxygenase
CHMO: Cyclohexanone monooxygenase
CHMO_*Acineto*_: Cyclohexanone monooxygenase from *Acinetobacter* sp. strain NCIMB 9871
CHMO_*Acineto*_-Mut: Engineered CHMO_*Acineto*_
CHMO_*Acineto*_-Mut-_*E*_: Engineered CHMO_*Acineto*_ expressed in *E. coli* BL21 (DE3)
CHMO_*Acineto*_-Mut-_*P*_: Engineered CHMO_*Acineto*_ expressed in *P. pastoris* X33
FAD: Flavin adenine dinucleotide
*GAP*: Glyceraldehyde-3-phosphate dehydrogenase promoter
NAD(P)H: Nicotinamide adenine dinucleotide (phosphate)
