# Supplementary material for: Secretory expression of cyclohexanone monooxygenase by methylotrophic yeast for efficient omeprazole sulfide bio-oxidation
Source: Bioresour Bioprocess. 2021 Aug 27;8(1):81. doi: 10.1186/s40643-021-00430-1 (PMC10992682; doi:10.1186/s40643-021-00430-1)
Supplement: Supplementary file 1 — Additional file 1: Table S1. Primers used in this study. Table S2. The cost of raw materials for CHMOAcineto-Mut preparation using different expression host. Figure S1. Shake flask fermentation activity of high-copy strain screening. Figure S2. Kinetic curves of CHMOAcineto-Mut-P and CHMOAcineto-Mut-E toward pyrmetazole (A) and NADPH (B). Figure S3. SDS-PAGE of deglycosylated CHMOAcineto-Mut by SpEndo H. Figure S4. Melting curves of CHMOAcineto-Mut-E and CHMOAcineto-Mut-P determined by ThermoFAD analysis. Figure S5. Photograph show of the ethyl acetate extraction of the esomeprazole in the aqueous phase of the reaction mixture (A) CHMOAcineto-Mut-E. (B) CHMOAcineto-Mut-P. Figure S6. Enzymatic oxidation reaction of pyrmetazole performed in 0.6 L scale and the isolation of esomeprazole sodium. Figure S7. Representative HPLC spectrum of FAD analysis of intracellular constituent. Figure S8. Representative HPLC spectrum of enzymatic esomeprazole synthesis. Figure S9. NMR spectrums of esomeprazole sodium salt. Figure S10. LR-MS spectrums of esomeprazole sodium salt. [file 40643_2021_430_MOESM1_ESM.docx]

*Supporting Information*

*for*

**Secretory Expression of Cyclohexanone Monooxygenase by Methylotrophic Yeast for Efficient Omeprazole Sulfide Bio-oxidation**

Ya-Jing Li, Yu-Cong Zheng, Qiang Geng, Feng Liu, Zhi-Jun Zhang, Jian-He Xu and Hui-Lei Yu*

State Key Laboratory of Bioreactor Engineering, Shanghai Collaborative Innovation Centre for Biomanufacturing, East China University of Science and Technology, Shanghai 200237, China. E-mail: huileiyu@ecust.edu.cn

**Table of the contents**

[Characterization of esomeprazole sodium salt S3](#_Toc73906551)

[Supplementary reference S3](#_Toc73906552)

[Supplementary tables S4](#_Toc73906553)

[Supplementary figures S6](#_Toc73906554)

[Supplementary HPLC spectrums S12](#_Toc73906555)

[Supplementary NMR spectrums S14](#_Toc73906556)

[Supplementary LR-MS spectrums S15](#_Toc73906557)

# Characterization of esomeprazole sodium salt


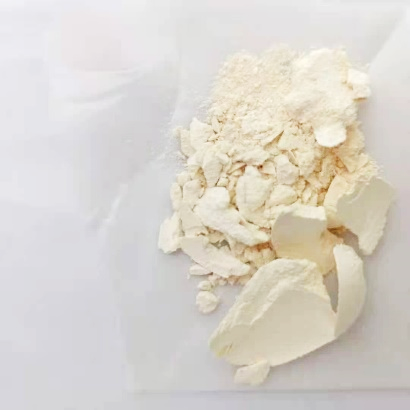
Esomeprazole sodium salt^[S1]^: 2.05 g, 34%. [α]30 D = +24 (c = 1.0 in H_2_O). *ee*>99%, {Lit: [α]20 D = +39 (c = 0.5 in H_2_O), *ee* = 99% of esomeprazole sodium salt [Jiang et al., 2009]} ^1^H NMR (600 MHz, DMSO-*d*_6_) δ/ppm: 8.23 (s, 1H), 7.32 (d, *J* = 8.7 Hz, 1H), 6.98 (d, *J* = 2.5 Hz, 1H), 6.55 (dd, *J* = 8.6, 2.5 Hz, 1H), 4.67 (d, *J* = 12.9 Hz, 1H), 4.38 (d, *J* = 12.9 Hz, 1H), 3.72 (s, 3H), 3.69 (s, 3H), 2.21 (s, 3H), 2.20 (s, 3H); ^13^C NMR (150 MHz, DMSO-*d*_6_) δ/ppm: 163.3, 161.7, 153.5, 152.0, 149.0, 146.9, 141.6, 126.5, 124.9, 117.4, 108.9, 99.4, 59.8, 59.7, 55.2, 12.9, 11.3. LRMS (EI-TOF): m/z calcd for C_17_H_18_N_3_SO_3_Na [M]: 367.1, found [M+H]^+^: 368.1

# Supplementary reference

[S1] Jiang B, Zhao XL, Dong JJ, Wang WJ (2009) Catalytic asymmetric oxidation of heteroaromatic sulfides with *tert*‐butyl hydroperoxide catalyzed by a titanium complex with a new chiral 1,2‐diphenylethane‐1,2‐diol ligand. *Eur J Org Chem* 2009: 987−991.

# Supplementary tables

Table S1. Primers used in this study.

| Name | Sequence |
| --- | --- |
| CHMO*_Acineto_*-Mut-*Eco*R I-FP | G**GAATTC**ATGAGCACCAAAATGGACTTCG |
| CHMO*_Acineto_*- Mut-*Not* I-RP | AAGGAAAAAA**GCGGCCGC**TTACGCGTTCGCTTGCTGCTT |
| CHMO*_Acineto_*- Mut-*Not* I-RP (his-tag) | AAGGAAAAAA**GCGGCCGC**CGCGTTCGCTTGCTGCTT |

Table S2. The cost of raw materials for CHMO*_Acineto_*-Mut preparation using different expression host.

|  | Strain | *P. pastoris* X33-pPICZαA-CHMO*_Acineto_*-Mut | | *E. coli* BL21(DE3)-pET-28(a)+-CHMO*_Acineto_*-Mut | |
| --- | --- | --- | --- | --- | --- |
| **Final fermentation volume (L)** | | 3 | | 3.3 | |
|  | Price  (CNY/kg) | Consumption  (g) | Cost  (CNY) | Consumption  (g) | Cost  (CNY) |
| Water | 0.005 | 4000 | 0.02 | 4000 | 0.02 |
| Peptone | 49 | 4 | 0.20 | - | - |
| Yeast extract | 28 | 4 | 0.11 | 19.5 | 0.55 |
| Tryptone | 30 | - | - | 19.5 | 0.59 |
| Glucose | 1.8 | 4 | 0.01 | - | - |
| IPTG | 8000 | - | - | 0.14 | 1.12 |
| Methanol | 2.3 | - | - | 1200 | 2.76 |
| Glycerol | 5 | 200 | 1 | 91 | 0.46 |
| Ammonia | 1.4 | 1000 | 1.4 | 250 | 0.35 |
| Na_2_SO_4_ | 0.7 | - | - | 21 | 0.01 |
| Na_2_HPO_4_ | 7.5 | - | - | 10.2 | 0.08 |
| MgSO_4_ | 3.5 | 29.8 | 0.10 | 0.75 | 0.003 |
| NH_4_Cl | 1.5 | - | - | 8.1 | 0.01 |
| Kanamycin | 20,000 | - | - | 0.015 | 0.30 |
| phosphoric acid (85%) | 4.7 | - | - | 53 | 0.25 |
| CaSO_4_ | 0.6 | 1.9 | 0.001 | - | 0 |
| K_2_SO_4_ | 3.8 | 36.4 | 0.14 | - | 0 |
| KOH | 2.5 | 8.3 | 0.02 | - | 0 |
| PTM_1_ trace salt | 1.25 | 16 | 0.02 | - | 0 |
| Antifoam | 20 | 1.5 | 0.03 | 1.5 | 0.03 |
| **CHMO preparation** | | | | | |
| K_2_CO_3_ | 8 | 53 | 0.42 | - | - |
| KH_2_PO_4_ | 7.5 | - | - | 61.2 | 0.46 |
| KOH | 2.5 | - | - | 20.4 | 0.05 |
| Water | 0.005 | 250 | 0.001 | 4500 | 0.02 |
| **Total** |  |  | 3.47 |  | 5.92 |

# Supplementary figures

**Figure S1**. Shake flask fermentation activity (1% methanol each day, v/v, induced for 72 h) of X33-pPICZαA-CHMO*_Acineto_*-Mut strain grown on YPDZ agar plates with different zeocin concentration.


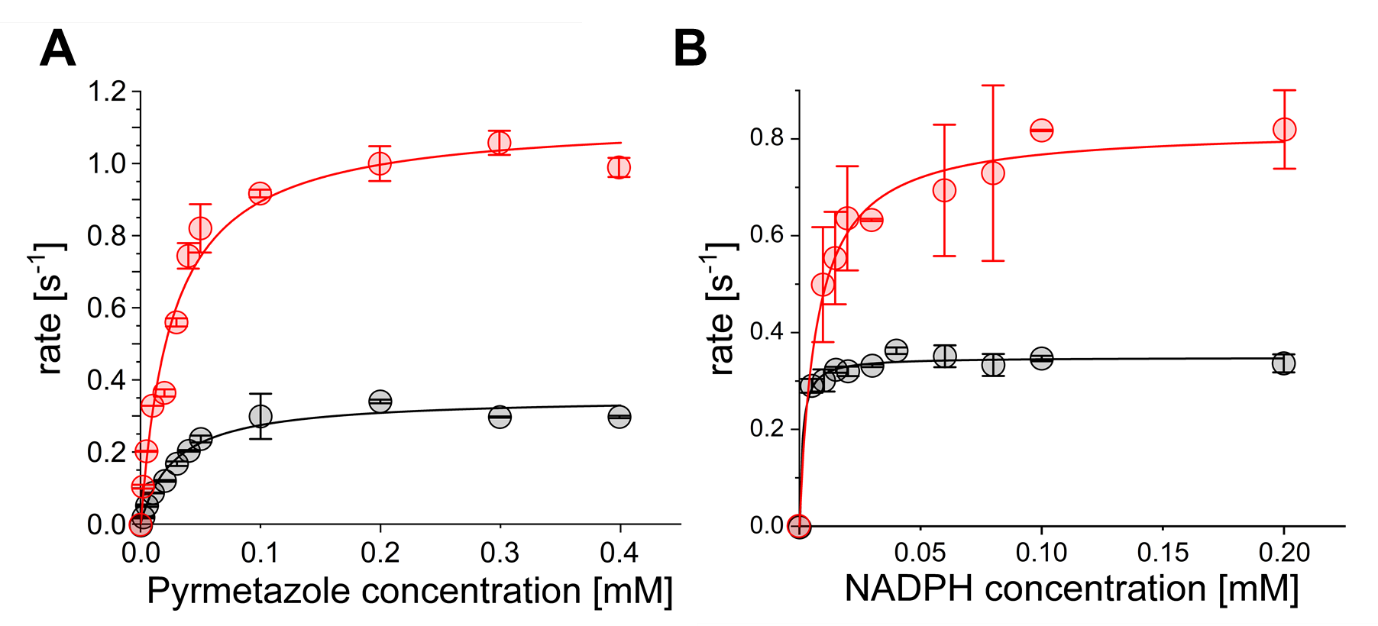


Figure S2. Kinetic curves of CHMO*_Acineto_-*Mut*-_P_* (**○**) and CHMO*_Acineto_-*Mut*-_E_* (**○**) toward pyrmetazole (A) and NADPH (B).


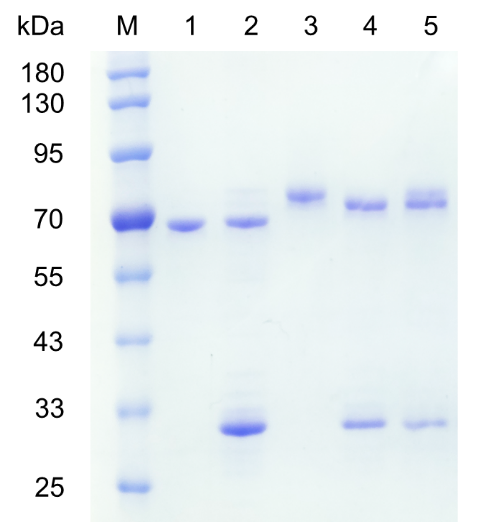


Figure S3. SDS-PAGE of deglycosylated CHMO*_Acineto_*-Mut by *Sp*Endo H. M: standard protein marker. Lane 1: purified CHMO*_Acineto_-*Mut*-_E_*; lane 2: purified CHMO*_Acineto_-*Mut*-_E_* after incubation with *Sp*Endo H; lane 3: purified CHMO*_Acineto_-*Mut*-_P_*; purified CHMO*_Acineto_-*Mut*-_P_* after incubation with *Sp*Endo H; lane 5: deglycosylated CHMO*_Acineto_-*Mut*-_P_* + native CHMO*_Acineto_-*Mut*-_P_*_._


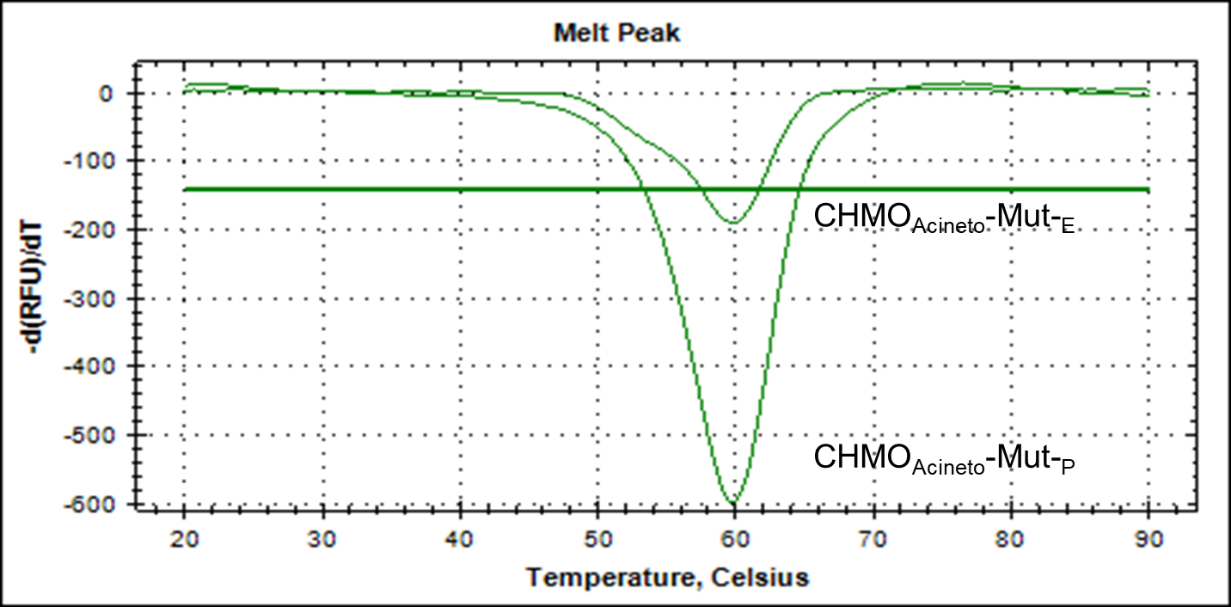


Figure S4. Melting curves of CHMO*_Acineto_-*Mut*-_E_* and CHMO*_Acineto_-*Mut*-_P_* determined by *Thermo*FAD analysis.


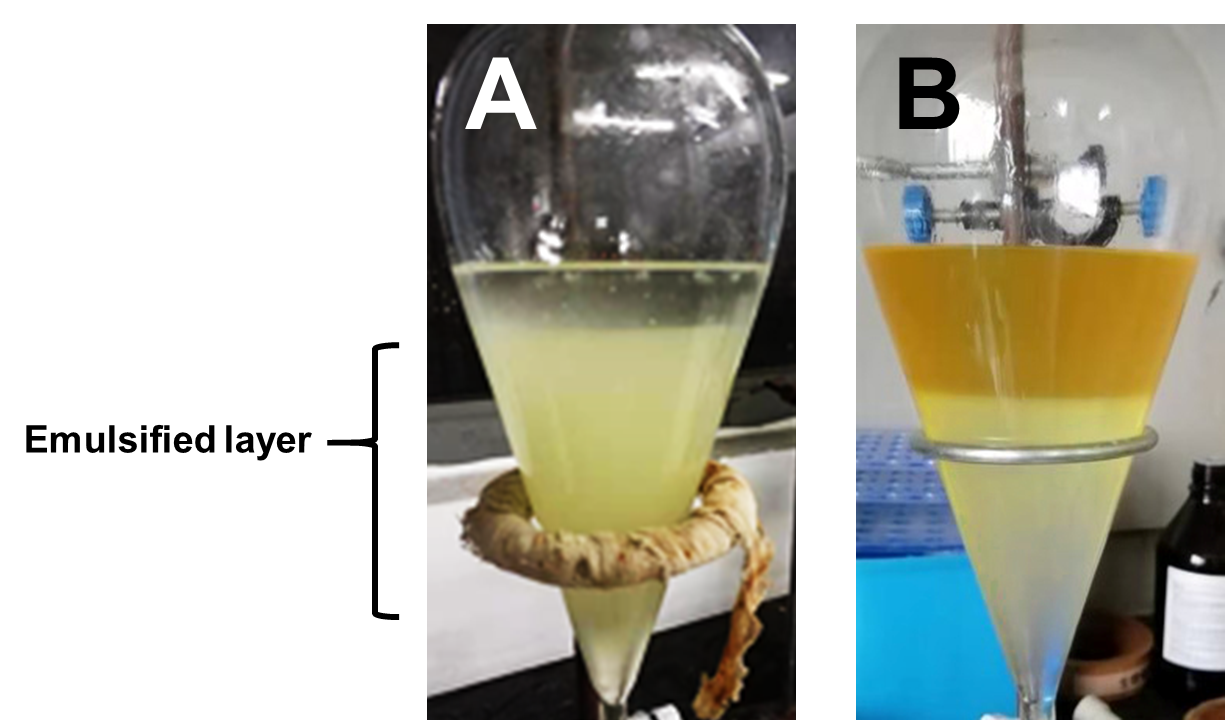


Figure S5. Photograph show of the ethyl acetate extraction of the esomeprazole in the aqueous phase of the reaction mixture (A) CHMO*_Acineto_-*Mut*-_E_*. (B) CHMO*_Acineto_-*Mut*-_P_*.

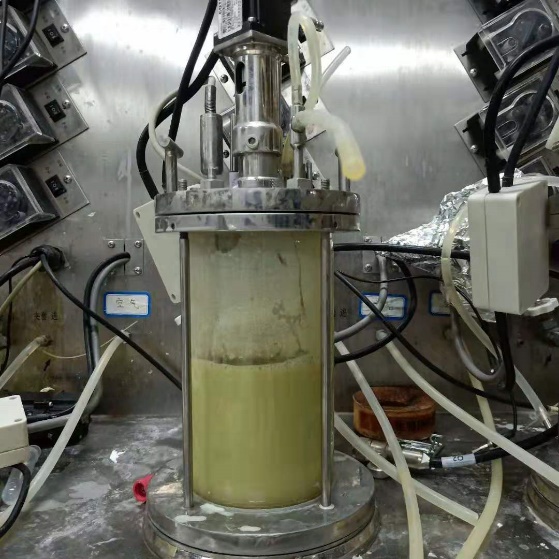


Figure S6. Enzymatic oxidation reaction of pyrmetazole performed in 0.6 L scale and the isolation of esomeprazole sodium.

# Supplementary HPLC spectrums


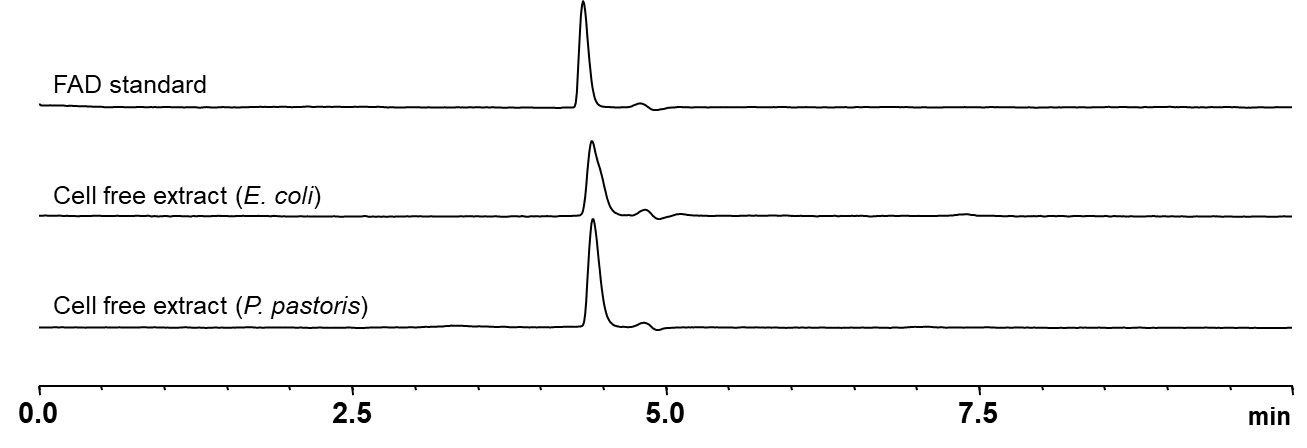


Figure S7. Representative HPLC spectrum of FAD analysis of intracellular constituent.


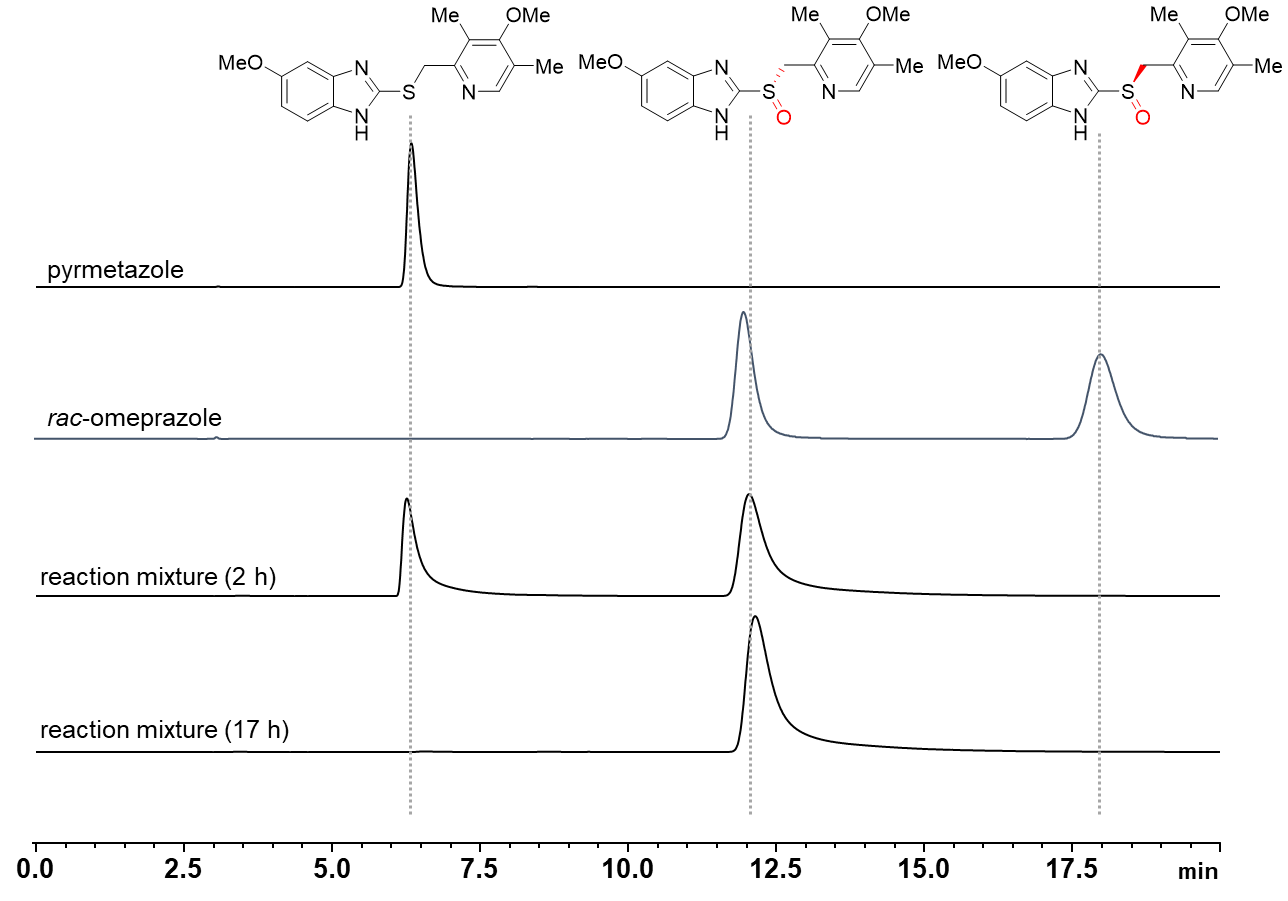


| Entry | Ret. Time [min] | | | Height [mV] | | | Area [mV∙s] | | | Area [%] | |
| --- | --- | --- | --- | --- | --- | --- | --- | --- | --- | --- | --- |
|  | Peak 1 | Peak 2 | Peak 3 | Peak 1 | Peak 2 | Peak 3 | Peak 1 | Peak 2 | Peak 3 | Peak 2 | Peak 3 |
| pyrmetazole | 6.339 | - | - | 131.916 | - | - | 1853.145 | - | - | - | - |
| *rac*-omeprazole | - | 11.978 | 18.016 | - | 114.149 | 75.523 | - | 2627.241 | 2599.665 | 50.264 | 49.736 |
| reaction mixture (2 h) | 6.258 | 12.040 | - | 534.482 | 558.278 | - | 12458.368 | 22720.756 | - | 100 | - |
| reaction mixture (17 h) | - | 12.144 |  | - | 900.307 | - | - | 36703.504 | - | 100 | - |

Figure S8. Representative HPLC spectrum of enzymatic esomeprazole synthesis.

# Supplementary NMR spectrums


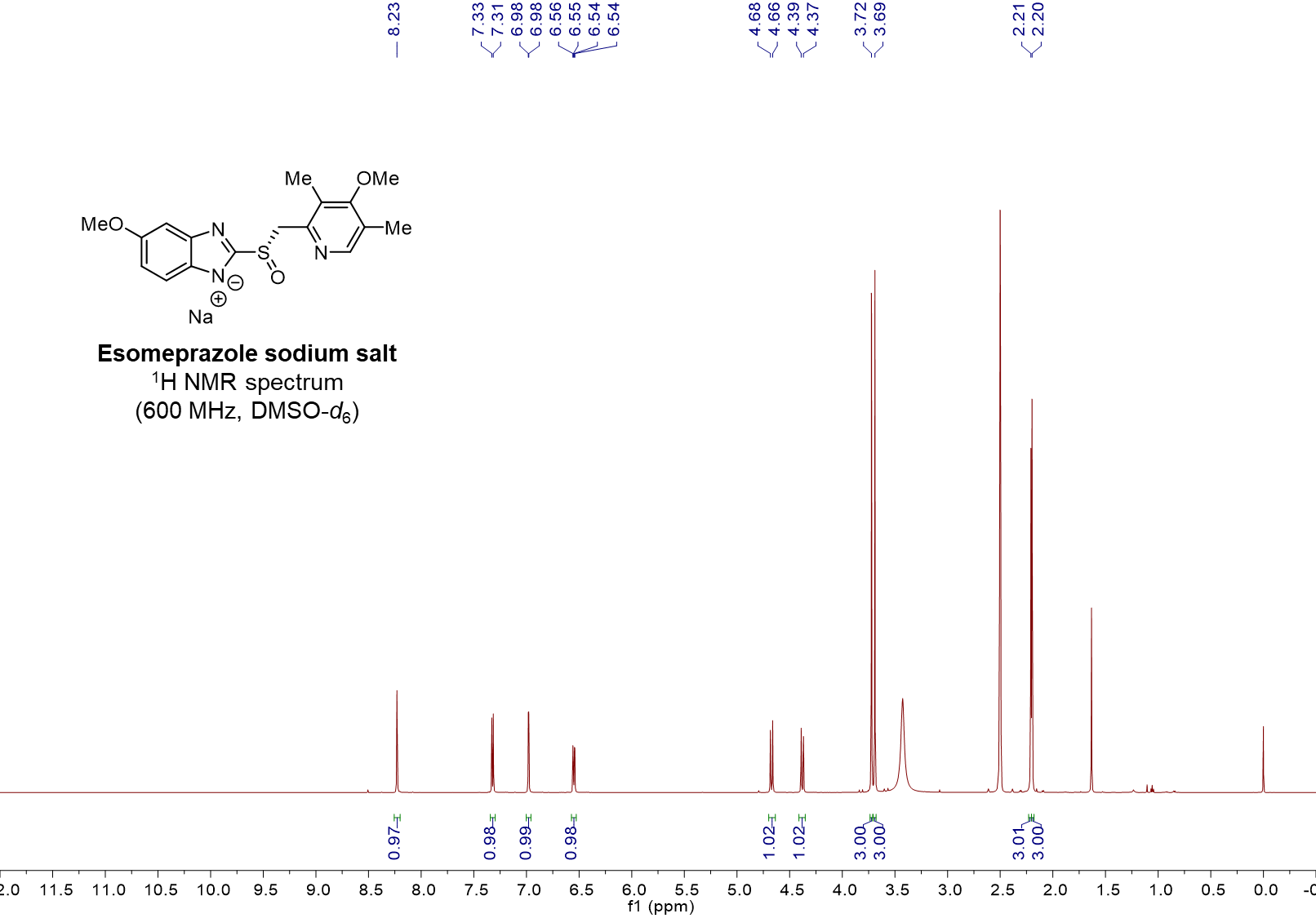


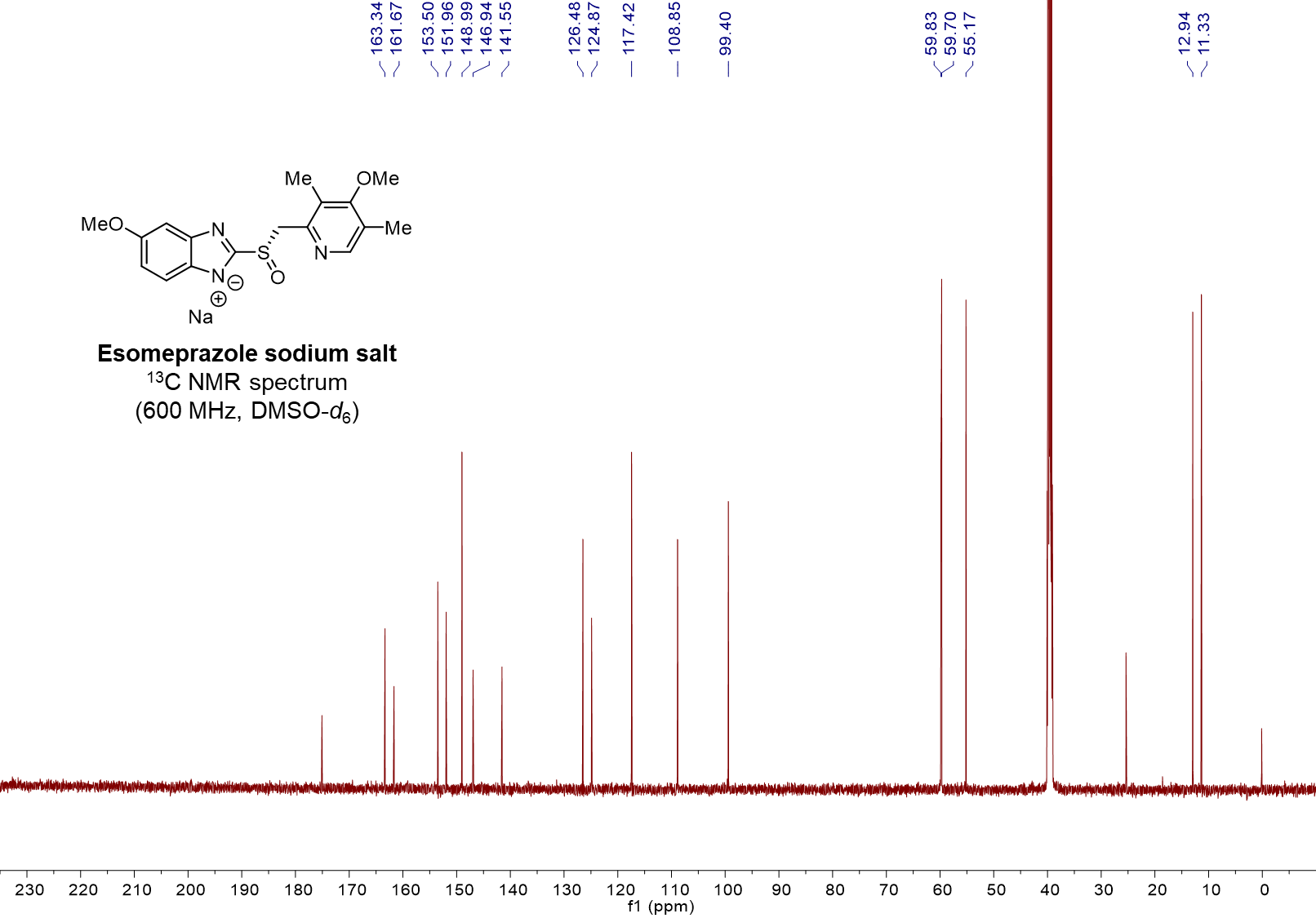


Figure S9. NMR spectrums of esomeprazole sodium salt.

# Supplementary LR-MS spectrums


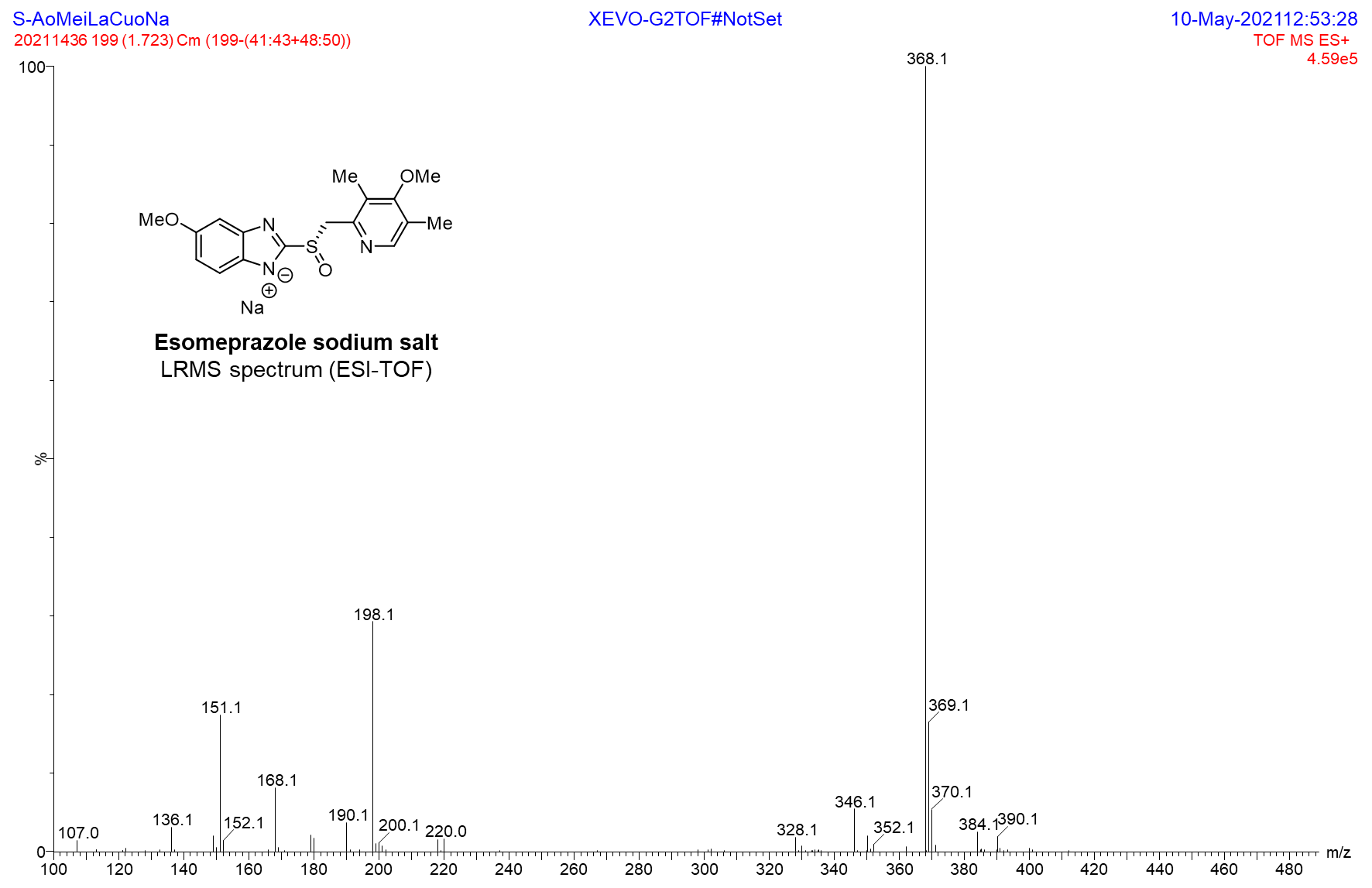


Figure S10. LR-MS spectrums of esomeprazole sodium salt.
